# Rheological and Nutritional Assessment of Dysphagia—Oriented New Food Preparations

**DOI:** 10.3390/foods10030663

**Published:** 2021-03-19

**Authors:** Francesca Cuomo, Martina Angelicola, Elisa De Arcangelis, Francesco Lopez, Maria Cristina Messia, Emanuele Marconi

**Affiliations:** Department of Agricultural, Environmental and Food Sciences (DiAAA), University of Molise, Via F. De Sanctis Snc, 86100 Campobasso, Italy; francesca.cuomo@unimol.it (F.C.); m.angelicola@studenti.unimol.it (M.A.); elisa.dearcangelis@unimol.it (E.D.A.); lopez@unimol.it (F.L.); marconi@unimol.it (E.M.)

**Keywords:** dysphagia, food supplement, energy and protein intakes, rheology

## Abstract

Dysphagia that involves difficulty swallowing food and liquids is a symptom of different diseases. In some cases, patients who experience this symptom should be fed with modified consistency foods. Dysphagia is often accompanied by malnutrition and dehydration and an interesting approach to dealing with these conditions is to provide patients with nutrient-rich foods. In this study, two new food formulations for dysphagia patients are proposed: a cereal-based protein meal and a vegetable cream. The nutritional and rheological characteristics of the two innovative preparations were assessed and compared with those of commercial products. The proposed protein meal formulations meet the criteria for the “high protein” claim and the vegetable cream meets those of the “source of fiber” claim. The rheological investigation revealed that the flow properties of the innovative formulations were comparable to those of the commercial ones. Based on these preliminary outcomes, this investigation represents an interesting perspective potentially valuable to enlarge the offer of possibilities for people suffering from swallowing disorders.

## 1. Introduction

Dysphagia is a medical disorder related with the difficulty of chewing and swallowing that can affect children, adults and the elderly [1,2,3,4,5]. It may derive from oropharyngeal or esophageal disorders related to functional causes, such as dementia and Parkinson’s disease, or to post-stroke conditions [6,7,8].

A diet with modified food consistency can, on some occasions, help people with problems of chewing and swallowing and, the right properties of density and viscosity should be found to facilitate their ingestion. The use of thickening agents that modify fluid flow behavior is crucial to regulate food consistency in order to slow down the transportation rate to reduce choking or other aspiration-correlated risks [9,10]. However, thickened food should also be pleasant to the taste. A number of thickeners are commercially available, like modified starches, xanthan gum, locust bean gum, gelatin, and carrageenan [10,11], and very often, they alter the sensorial properties of foods. Recognizing which type of thickener provides the best or the worst performance depends on several factors, and mainly on the consumer’s different choosing criteria and personal preferences [12].

Patients with dysphagia very often undergo malnutrition and dehydration, also as a consequence of food intake limiting. For these reasons, it is important to provide meals with adequate intake of nutrients to meet the patients’ nutritional requirements. Food characteristics for dysphagia patients should be personalized in order to guarantee an adequate food program that covers nutritional needs without ignoring sensorial acceptability [5,13]. According to the individual needs, the design of formulations that can be easily prepared at home with common food ingredients available in non-specialized shops remains a desirable goal [14].

Different methods exist to measure the flow characteristics of thickened drinks and food for dysphagia management, among them, there are the test of elongation flow and the line spread test [6,10,15], and the rheological tests. The parameters considered in the sample rheology evaluation deal with the flow curve behavior and the viscoelastic characteristics, defined by rotational and oscillatory tests, respectively [16,17]. Recently, Sharma and colleagues [18], evaluated the effects of different hydrocolloids on pureed carrots considering rheological, textural, and sensorial properties. Cho and coworkers [19] considered the rheological properties of thickened beverages for dysphagia patients like fruit juices, milk, and sport drinks prepared with four xanthan gum-based thickeners.

In this investigation, the rheological aspects of commercial products for dysphagia patients and two new dysphagia-oriented products were assessed through rotational and oscillatory tests. A protein meal based on cereal flours and a vegetable cream were formulated considering the nutritional aspects and compared to commercial products. The final aim of the present study was thus joining the nutritional value with texture modification that made the new formulations similar to the commercial products. This study represents an early stage in the design of formulations for people with swallowing difficulties.

## 2. Materials and Methods

### 2.1. Materials

Commercially available products for dysphagia patients, such as AM gel cup (gelled water fruit aroma, AG), high-protein powder supplement (Delical Cereal Instant, DCI), and dessert cream (Delical Cream Dessert, DCD) were from DMF srl (Dietetic Metabolic Food s.r.l, Limbiate, Italy). The DCI powder was dispersed in water according to the manufacturer instructions before the use (50 g of powder dispersed in 150 mL of warm or cold water), while AG and DCD were used as is. Patients with dysphagia are used to consume AG as a substitute for water, DCI as a protein meal to be prepared at the time of use, and DCD as dessert. Nutritional facts of AG, DCI, and DCD (as declared by manufacturers) are shown in Table 1.

Instant emmer and rice flours were from Alce Nero (Bruno, AT, Italy), concentrated hydrolyzed instant whey proteins were from Farmalabor Srl, (Canosa di Puglia, BT, Italy) and pregelatinized maize starch was from Unilever (Rome, Italy). Chard and lentils were purchased from a local supermarket. All chemicals and solvents used were of analytical grade and purchased from Carlo Erba (Milan, Italy) and from Sigma-Aldrich Srl (Milan, Italy).

The two formulations were prepared in a laboratory by mixing the ingredients with a thickening agent (gelatinized maize starch). In particular, the protein meal (PM) was prepared by mixing instant rice and emmer flours with dried whey protein and gelatinized maize starch. The vegetable cream (VC) was prepared by mixing steam-cooked and blended vegetables (fresh chard and dried lentils) with the gelatinized maize starch. In order to obtain the right consistency of preparations, PM was prepared, adding warm water (~50 °C) to ingredients (see Table 2), fixing the following 1:2.5 weight ratio, while VC was prepared by mixing vegetables and water to a 1:1 weight ratio. Visual appearance of PM and VC is reported in Figure S1A,B (Supplementary Material), respectively.

### 2.2. Chemical Composition Analyses

Protein meal (PM) and vegetable cream (VC) were analyzed for moisture, ash, fat (determined on dried samples with solvent extraction method), and protein (N × 6.25) content, according to standard methods 925.09, 923.03, 991.36, and 984.13 of AOAC [20]. Total dietary fiber content was determined according to AACC Method 32.05 [21].

### 2.3. Essential Amino Acids Analysis

Acidic hydrolysis was carried out to analyze amino acid content [22]. An aliquot of sample, corresponding to 25 mg of protein and 25 mL of 6 N HCl, was placed in a pyrex tube in which the vacuum was made before being placed in the oven at 110 °C for 24 h. Afterward, the sample was cooled and filtered. Tryptophan analysis was carried out after sample alkaline hydrolysis. Briefly, a sample containing 10 mg of protein was placed in a pyrex glass tube containing 1 mL of distilled water. After shaking, 5 mL of 10 N NaOH and 4 mL of distilled water were added. The sample was hydrolyzed for 18 h at 110 °C. After cooling, the sample was neutralized by adding 6 N HCI drop by drop. At the end of acidic and alkaline hydrolysis, the samples were dried and re-dissolved in 0.1 N HCl. Before analysis, all samples were diluted 1:50–1:100 with ultra-pure water, filtered through 0.20 μm filter, and then injected in the ICS6000 chromatographic system (Thermo Fisher Scientific S.p.A, Milano, Italy). Conditions for chromatographic separation of amino acids are reported in Table S1 (Supplementary Material). Quantification was carried out by means of an external amino acid standard mix (Sigma Chemical Co., St. Louis, MO, USA). Standard calibration curves were made (0.5 and 100 μM) to define the system linearity and the limits of detection and quantification.

Chemical Score (CS) was calculated using the equation, which compares the content of each essential amino acid (EAA) of the test protein to the amount of the same amino acid of the reference Food and Agriculture Organization pattern [23].

### 2.4. Rheological Characterization

The rheometer, Haake MARS III (Thermo Scientific, Karlsruhe, Germany), was used for the rheological characterization. All measurements were made at 25 °C, and the temperature was controlled by a Peltier module TM-PE-P coupled with a liquid bath module (Phoenix II, Thermo Scientific, Karlsruhe, Germany). The samples (2.9 mL) were poured onto the surface of the lower plate, and the upper plate was lowered to 1 mm gap distance. Before testing, samples were left equilibrating for 10 min to allow for mechanical and temperature equilibrium.

Flow curves and thixotropic behavior were studied through rotational tests. Both tests were realized in controlled rate mode (CR). Thixotropy curves were obtained through hysteresis loop experiments carried out in three steps: (1) Upward curve was made by varying the shear rate from 0 to 100 s^−1^ in 100 s, (2) plateau curve at the maximum shear rate (100 s^−1^) for 30 s, (3) downward curve by varying the shear rate from 100 to 0 s^−1^ in 100 s.

Oscillatory dynamic tests: The range of linear viscoelasticity (LVE) was determined through amplitude sweep test at 1 Hz of frequency, and frequency sweep test was made in controlled deformation and a frequency range from 0.01 to 10 Hz.

### 2.5. Statistical Analysis

Data reported for all parameters are the average values of measurements obtained from the analysis of three different aliquots of each sample and were expressed as mean ± standard deviation (mean value ± sd).

## 3. Results

### 3.1. Nutritional Aspects of Innovative Formulations

Different formulations were studied using instant commercial flours and vegetables to obtain new products such as a Protein Meal (PM) and a Vegetable Cream (VC) suitable for patients affected by dysphagia disorder. Different raw materials were combined to obtain new suitable products with adequate nutritional values and the selected formulations provided in this study are shown in Table 2.

The recommended daily dose of protein for an adult was taken into account to design the PM formulation, specifically 0.8 g/kg of body weight [24]. In view of this, we recall that a man of 80 kg should ingest around 60 g of protein/day distributed over three main meals, each providing no less than 20 g of protein, to get the right amount of protein per day.

In an attempt to give an alternative formulation, instant rice and emmer flours were coupled to produce a cereal-based meal to which gelatinized maize starch was added as a thickening agent. With the idea to formulate a product as natural as possible, no other emulsifier or stabilizer was used. In order to obtain a preparation with a high nutritional value and a pleasant flavor, the legume flours generally used in this type of food preparation and whose flavor is a major factor limiting its use [25] were replaced with dried whey proteins. We recall here that whey proteins represent a source of proteins with a high biological value that are important for the health and functions of the body, especially for children, in sports nutrition, and for dysphagia patients [26].

Table 3 shows the chemical composition and the energy values of PM and VC. The chemical analysis highlighted that the protein content of PM was 21.9 g/100 g, and therefore, it represented a meal that could be intended for dysphagia patients for its high protein intake. According to EC Regulation 1924/2006 on nutrition and health claims on food products [27], the PM formulation responded to the requisites for the claim “high protein” because 20% of the energy value is provided by proteins. Moreover, the energy value of 1573 kJ/371 kcal was close to that found in commercial preparation of DCI, with the advantage of the absence of foreign fats or glucose syrups, generally present in these types of formulations.

The main factor that determines the quality of proteins is the so-called amino acid pattern. The amino acid that has the lowest chemical score is defined as the limiting amino acid and the lysine with its lowest score is the limiting amino acid for cereals like rice and emmer. The PM produced in this investigation, including high-quality whey proteins, showed an improved CS value of 100, instead of 72 and 44 CS values of rice and emmer flours, respectively (Table 4). These data denote a high protein quality of the innovative formulation, and therefore, its ability to supply to the dysphagia patient the optimum amount of amino acids. Moreover, the here obtained PM formulation also manages to meet the requirements of UE Regulation No. 2016/1413 [28], regarding the composition of proteins for a complete meal replacement for weight control because its CS was greater than 80. The products made and analyzed in this study seem to have the feature for a more natural and easy make-ahead meal.

On the other hand, it should be necessary to highlight that a suitable and appropriate diet is essential for patients with dysphagia to ensure the daily nutritional intake in a varied and balanced way by providing adequate amounts of proteins, fats, carbohydrates, and fibers. VC was, therefore, formulated because currently, there are no vegetable-based products on the Italian market for patients with dysphagia. It is expected that a vegetable cream contains an adequate fiber content. Dietary fiber is represented by a variety of molecules with different chemical-physical properties that correspond to different physiological properties [25]. Fibers are resistant to hydrolysis and absorption in the small intestine and reach the colon substantially unchanged. Consequently, dietary fiber plays an indispensable role in regulating intestinal function by increasing fecal mass and accelerating intestinal transit time. VC preparation showed (see Table 3) a total dietary fiber content of 3.2 g/100 g. According to EC Regulation 1924/2006 [28], this type of cream can be defined as a “source of fiber” because it contains at least 3 g of fiber per 100 g of product. This type of product could be used as a side dish or as an alternative to sugary puddings, useful to provide an adequate intake of dietary fiber.

### 3.2. Rheological Behavior: Rotational Tests

**Figure 1:**
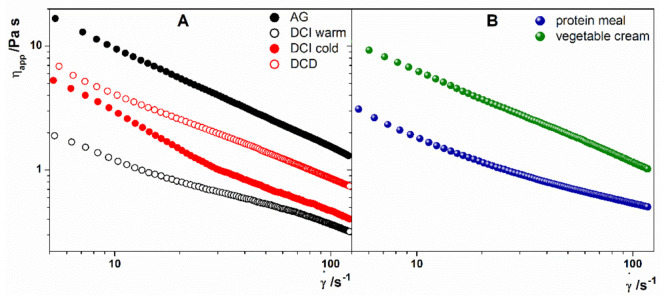
(**A**) Flow curves of AG (full black circles), DCI hydrated with warm water (open black circles), DCI hydrated with cold water (full red circles), DCD (open red circles), (**B**) flow curves of Protein Meal (full blue circles) and Vegetable Cream (full green circles).

The rheological behavior of commercial and innovative preparations was studied by rotational and oscillatory tests. Data of the flow curves were fitted to the Herschel-Bulkley model expressed by the following equation:

(1) $$\tau =\tau_{0}+k\dot{\gamma }{\;}^{n}\;$$

where *τ* is the shear stress (Pa), *τ*_0_ is the yield stress (Pa), *k* is the consistency index (Pa s^n^) that reflects the fluid consistency, $\dot{\gamma }$ is the shear rate (s^−1^) and *n* (dimensionless parameter) is the index of rheological behavior. For *n* = 1 the fluid is Newtonian, for *n* < 1 the fluid is considered shear-thinning and for *n* > 1 is shear-thickening [29,30]. The data extrapolated by the fitting to Equation (1) are reported in Table S2 (Supplementary Material). From the data, it emerges that all the formulations, commercial or new, are characterized by a yield stress with the exception of DCI warm, whose value of *τ*_0_ is close to 0, and, from the behavior index *n*, that all fluids are non-Newtonian shear-thinning fluids. Since shear stress and shear rate were not correlated by a linear relationship, apparent viscosity (*η_app_*) of the fluid decreased with the shear rate increase, as illustrated in Figure 1. The shear rate dependence of *η_app_* for AG, DCI hydrated with warm (60 °C) or cold (15 °C) water and DCD is illustrated by Figure 1A, that for the two new formulations, is reported in Figure 1B. The shear-thinning behavior of the fluids is explainable with the fluid structure modification with the applied shear rate. If the fluid contains particles oriented randomly at rest, they will orient parallel to the fluid flow during shearing. If the fluid contains suspended agglomerated particles, the agglomerates are disassembled into primary particles during shearing, thus opposing a lower resistance to the generated flow. Considering the innovative formulations, as expected, the apparent viscosity values resulted higher in VC than in PM because the former was formulated with a higher amount of thickener (gelatinized maize starch) than the latter.

According to the apparent viscosity values measured at a shear rate of 50 s^−1^ meal preparations for patients with dysphagia can be classified in thin-, nectar-, spoon-, and honey-like fluids [31,32,33].

In particular, for 1< *η_app_* < 0.050 Pas as thin-like fluids, for 0.051< *η_app_* < 0.350 Pas as nectar-thick fluids, for 0.351< *η_app_* < 1.75 Pas as honey-thick, and for *η_app_* > 1.750 Pas as spoon-thick.

The categories in which the commercial and the lab-made formulations fell, according to the *η_app_* measured at 50 s^−1^ are reported in Table 5. As the table shows, the new formulations are spoon-like preparations, like AG having *η_app_* values higher than 1.75 Pa s.

**Figure 2:**
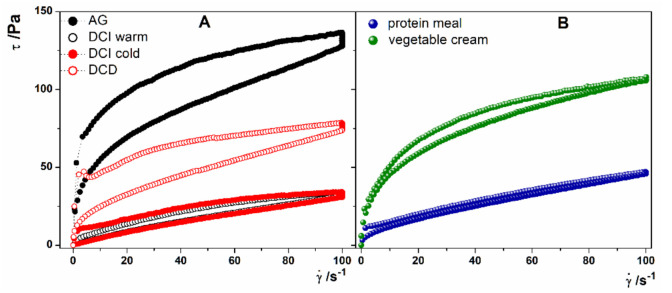
Thixotropy loop experiments of (**A**) AG (full black circles), DCI hydrated with warm water (open black circles), DCI hydrated with cold water (full red circles), DCD (open red circles), (**B**) Protein Meal (full blue circles) and Vegetable Cream (full green circles).

With the aim to compare the commercial preparations with the new formulations proposed, a further aspect has been considered to evaluate the thixotropic behavior. This characteristic can be studied by rotational loop experiments at a controlled rate. The loop is given by shear stress response registered at increasing and decreasing shear rate down to zero. Thixotropic materials are characterized by the hysteresis area determined as the difference in the area defined by the upward and the backward curve that represents the energy to breakdown the fluid structure. The greater is the hysteresis area, the longer is the time required by the fluid to recover its structure. According to the experimental results shown in Figure 2A, the tested products were all thixotropic fluids. Among the commercial products, AG had higher thixotropic behavior, followed by DCD and finally by the two DCI formulations with the smaller thixotropic area. Considering the two new formulations reported in Figure 2B, it is possible to deduce that these meal preparations behave in a similar way compared with the commercial products, although these exhibit a less marked thixotropic behavior.

### 3.3. Rheological Behavior: Oscillatory Tests

**Figure 3:**
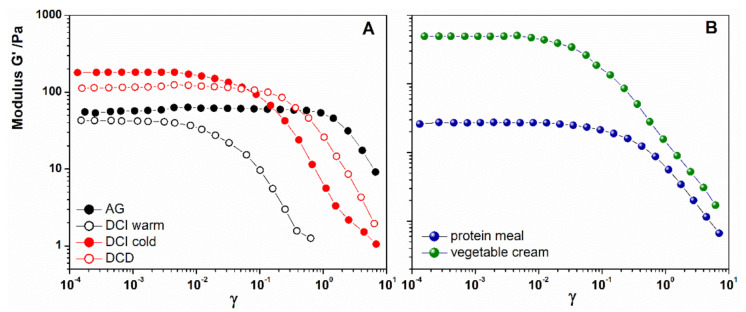
(**A**) Amplitude strain sweep of AG (full black circles), DCI hydrated with warm water (open black circles), DCI hydrated with cold water (full red circles), DCD (open red circles), (**B**) amplitude strain sweep of Protein Meal (full blue circles) and Vegetable Cream (full green circles).

**Figure 4:**
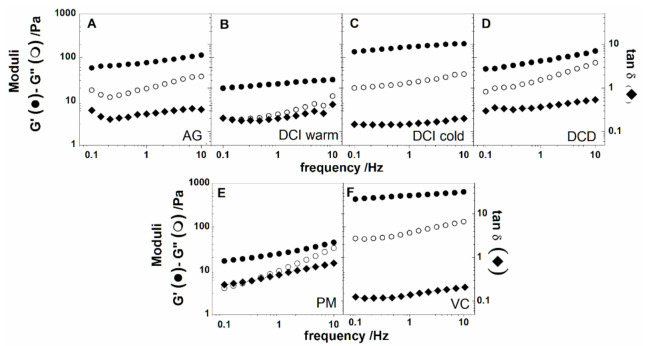
Frequency sweep for AG (**A**), DCI hydrated with warm water (**B**), DCI hydrated with cold water (**C**), DCD (**D**), Protein Meal (**E**), Vegetable Cream (**F**). G’ modulus is represented with full circles, G” with open circles and the loss tangent with full diamonds.

The results of the oscillation tests provided significant evidence on the sample structure. Strain sweep tests (Figure 3) together with frequency sweep tests (Figure 4) revealed the gel-like behavior for all the samples that were characterized by the elastic modulus G’ higher than the viscous modulus G”. The linear viscoelastic (LVE) region for each sample was identified in Figure 3 as the region where G’ was independent of the applied deformation. It can be noted (Figure 3A) that the AG sample showed a longer range of linearity compared to the others. A slightly shorter linear region than AG was detectible for DCD, and higher deformation dependence (smaller LVE) was registered for DCI, in particular if hydrated with warm water.

The mechanical strength of the samples evaluated by comparing the values of G’ in the LVE region [34], resulted higher for DCI hydrated with cold water, followed by the cream dessert, DCD, AG, and finally by DCI hydrated with warm water. The difference detected between DCI warm, and DCI cold can be attributed to the different degree of ingredient hydration provided by the different temperature. At 60 °C, indeed, the ingredients are better solubilized, and the G’ resulted lower than that of DCI cold, hydrated at 15 °C. Here, it is important to remark that in spite of the small differences observed with the rotational test, a marked variation has been detected in the mechanical spectra [18,35].

Considering the new formulations, in Figure 3B, PM exhibited a lower strength than the VC but a longer LVE. The vegetable cream, on the other hand, had higher rigidity (G’) because it contained a higher thickener concentration [17,19].

Frequency sweep tests, performed within the linear viscoelastic region of each sample, enabled to determine the fluid dependence from the frequency in the range between 0.1 and 10 Hz. Figure 4A–F showed the outcomes of the test.

The low frequency simulates the behavior at long-term (slow motion) while the high-frequency region corresponds to the behavior at short-term (rapid motion). All the samples, as can be seen from the figures, presented the elastic modulus (G’) higher than the viscous modulus (G”) in the frequency range explored, indicating that the solid-like character prevails when a load is applied. Moreover, a very low dependence of the elastic modulus from frequency is found for all the samples except for DCD that seemed more frequency-dependent compared to the other samples. On the other hand, G” modulus showed certain dependence from frequency, but for DCI hydrated with cold water, denoting that for these samples in the short-term behavior, the viscous character influenced the response of the material. Nevertheless, by checking the loss tangent values, *tan δ*, and considering they remained lower than unity, they indicated the prevalence of the elastic behavior in the frequency range applied.

In the new proposed formulations, the mechanical response to the frequency sweep test of the protein meal is very similar to that of DCD, while the response of the VC resembles that of DCI hydrated with cold water. Moreover, the values of the tangent loss (*tan δ*) of the new formulations were similar to DCD for PM formulation and similar to the DCI (cold) for VC. The value of *tan δ* has been identified as a criterion to classify a fluid as safe to swallow for dysphagia patients by Ishihara et al. [36]. The same criterion has been adopted very recently by Talens et al. [35] in a study regarding the formulation of thickened pea cream for people with swallowing problems. The criterion identifies the values of *tan δ* in the range 0.1–1 for fluids suitable for dysphagia disorders. Then, since *tan δ* is given by the ratio G’’/G’, the same holds if G’’ is one order of magnitude lower than G’ or of the same order of magnitude. Considering our samples, either the new formulations, PM and VC, or the commercial formulations met the criterion described.

On the whole, this research has provided a comparison of the nutritional and the rheological characteristics between commercial solutions for people with swallowing difficulties and the new formulations we proposed. Based on the recent literature on this issue [37], we are aware that the outcomes of our study should face the clinical aspects for a safe administration of the proposed innovative products to people suffering from swallowing complications. Moreover, it would be desirable that in identifying the impact of food thickening on the reduction of penetration and aspiration, some objective parameters are defined to allow researchers to formulate food preparations, ensuring that even thicker foodstuffs could be easily swallowed.

## 4. Conclusions

In this investigation, two dysphagia-oriented formulations have been proposed, one rich in protein (protein meal) and the other based on vegetables (vegetable cream). The nutritional aspects evaluated on the new formulations revealed that the protein content in the protein meal was 21.9% and the fiber content in the vegetable cream was 3.2%. These values allowed reaching the claim “high protein” for the protein meal and “source of fiber”, for the vegetable cream. The nutritional value of proteins in the protein meal assessed by the Chemical Score was high (CS = 100).

By comparing the rheological characteristics of the new formulations with those of commercial formulations for dysphagia, it emerged that they were comparable for the rheological behavior.

Based on these results, this investigation can be seen as a starting point toward the design of further alternatives for consumers with swallowing disorders. Moreover, considering the ingredients used and the procedure to prepare protein meal and vegetable cream, the proposed formulations could be prepared by non-specialized personnel in conditions of domestic use. To finally consider the proposed preparations suitable for administration to patients with swallowing difficulties, some other tests should be carried out. Their behavior during swallowing and sensory analysis carried out by trained people and/or human subjects have to be considered as important further steps.

## Figures and Tables

**Table 1:** Nutrition facts (g/100 g of product) of commercial preparations.

	AM Gel (AG)	Delical Cereal Instant (DCI)	Delical Cream Dessert (DCD)
Energy	15 kJ/4 kcal	1687 kJ/400 kcal	635 kJ/150 kcal
Fat, total	<0.5 g	9.9 g	4.9 g
-saturated	0.0 g	1.5 g	0.3 g
Carbohydrate	<0.5 g	57.6 g	16.7 g
-sugar	0.0 g	19.5 g	12.2 g
Fiber	1.2 g	6.0 g	0.0 g
Protein	<0.5 g	20.1 g	10.0 g
Salt	0.07 g	0.83 g	0.1 g

**Table 2:** Formulations proposed for Protein Meal (PM) and Vegetable Cream (VC).

Ingredients (%)	PM	VC
Instant rice flour	65.5	0
Instant emmer flour	10	0
Dried whey protein	20	0
Gelatinized maize starch	4.5	8
Chard puree	0	37
Pureed lentils	0	55

**Table 3:** Chemical composition (g/100 g) and energy value of innovative protein meal (PM) and vegetable cream (VC).

	PM	VC
Moisture	6.7 ± 0.05	85.0 ± 0.02
Fat	1.6 ± 0.02	0.3 ± 0.01
Carbohydrate *	66.3	17.6
Fiber	1.8 ± 0.10	3.20 ± 0.08
Protein	21.9 ± 0.12	4.1 ± 0.05
Ash	1.65 ± 0.04	0.74 ± 0.05
Energy kJ/kcal	1573/371	405/94

* Calculated by difference.

**Table 4:** Essential Amino Acid (g/100 g protein), Chemical Score (CS) and limiting amino-acid of rice flour, emmer flour, whey proteins powder, and Protein Meal (PM).

Essential Amino Acid	Rice Flour	Emmer Flour	Whey Proteins Powder	Protein Meal (PM)
Histidine	2.50 ± 0.02	2.11 ± 0.01	2.13 ± 0.03	1.96 ± 0.05
Isoleucine	4.10 ± 0.05	3.42 ± 0.02	7.12 ± 0.10	5.89 ± 0.10
Leucine	8.20 ± 0.10	6.80 ± 0.05	11.35 ± 0.55	9.83 ± 0.42
Lysine	3.48 ± 0.23	2.13 ± 0.03	9.83 ± 0.13	7.49 ± 0.10
Methionine	2.42 ± 0.00	1.58 ± 0.02	2.25 ± 0.09	2.18 ± 0.19
Cystine	1.80 ± 0.21	2.00 ± 0.10	2.51 ± 0.21	2.13 ± 0.15
Phenylalanine	5.33 ± 0.11	4.89 ± 0.10	3.44 ± 0.28	3.76 ± 0.05
Tyrosine	5.28 ± 0.08	2.56 ± 0.04	3.42 ± 0.56	3.41 ± 0.22
Threonine	3.53 ± 0.21	2.81 ± 0.00	7.72 ± 0.02	5.80 ± 0.00
Valine	5.85 ± 0.02	4.14 ± 0.04	6.65 ± 0.23	5.65 ± 0.32
Tryptophan	1.21 ± 0.42	1.00 ± 0.04	1.93 ± 0.04	1.34 ± 0.10
Chemical Score (CS)	77	44	100	100
Limiting amino acid	Lysine	Lysine	-	-

**Table 5:** Apparent viscosity values measured at $\dot{\gamma }$ = 50 s^−1^ and formulation category for commercial and new preparations.

Formulation	η_app_/Pa s	Category
AG	2.60	Spoon-like
DCI warm	0.42	Honey-like
DCI cold	0.84	Honey-like
DCD	1.17	Honey-like
PM	1.90	Spoon-like
VC	3.30	Spoon-like

## Data Availability

The data presented in this study are available on request from the corresponding author.

## Supplementary Materials

The following are available online at https://www.mdpi.com/2304-8158/10/3/663/s1, Figure S1: Visual appearance of Protein Meal (A) and Vegetable Cream (B) preparations; Table S1: Conditions for chromatographic separation of amino acids carried out through ICS6000 chromatographic system; Table S2: Values of yield stress (τ_o_) consistency index (k), rheological behavior index (n) and correlation coefficient (R^2^), of commercial (AG, DCI warm, DCI cold and DCD) and innovative formulations (PM and VC) obtained from data fitting to Herschel-Bulkley equation.
