# Supplementary material for: Rheological and Nutritional Assessment of Dysphagia—Oriented New Food Preparations
Source: Foods. 2021 Mar 19;10(3):663. doi: 10.3390/foods10030663 (PMC8003745; doi:10.3390/foods10030663)
Supplement: Supplementary file 1 [file foods-10-00663-s001.pdf]

Rheological and Nutritional Assessment of Dysphagia – Oriented New Food Preparations

Francesca Cuomo [1], Martina Angelicola [1], Elisa De Arcangelis [1], Francesco Lopez [1], Maria Cristina Messia [1]*, Emanuele Marconi [1]

[1] Department of Agricultural, Environmental and Food Sciences (DiAAA), University of Molise, Via F. De Sanctis snc – 86100 Campobasso, Italy; francesca.cuomo@unimol.it (F.C.) ; m.angelicola@studenti.unimol.it (M.A.); elisa.dearcangelis@unimol.it (E.D.A.); lopez@unimol.it (F.L.), marconi@unimol.it (E.M.)
* Correspondence: messia@unimol.it (M.C.M.);

Supplementary materials

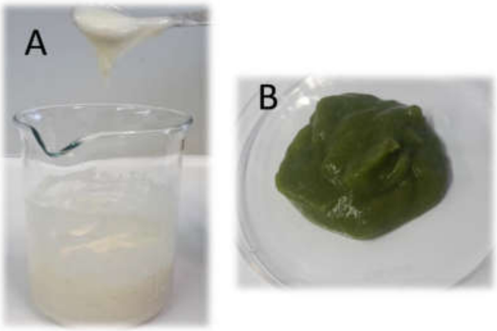

Figure S1: Visual appearance of Protein Meal (A) and Vegetable Cream (B) preparations.

Table S1: Conditions for chromatographic separation of amino acids carried out through ICS6000 chromatographic system.

Mobile Phase (0.250 mL/min)					Time/Potential waveform		
**Time (min)**	**H_2O (%)**	**NaOH (%)**	**NaOAc (%)**		**Time (sec)**	**Potential (V)**	**Integration**
0.0	80	20	0		0.00	+ 0.13	
2.0	80	20	0		0.04	+ 0.13	
12.0	80	20	0		0.05	+ 0.28	
16.0	68	32	0		0.11	+ 0.28	began
24.0	36	24	40		0.12	+ 0.60	
40.0	36	24	40		0.41	+ 0.60	
40.1	20	80	0		0.42	+ 0.28	
42.1	20	80	0		0.56	+ 0.28	end
42.2	80	20	0		0.57	− 1.67	
62.0	80	20	0		0.58	− 1.67	
					0.59	+ 0.93	
					0.60	+ 0.13	

Table S2: Values of yield stress (τ_o) consistency index (k), rheological behavior index (n) and correlation coefficient (R2), of commercial (AG, DCI warm, DCI cold and DCD) and innovative formulations (PM and VC) obtained from data fitting to Herschel-Bulkley equation: $\tau = \tau_0 + k\dot{\gamma}^{\,n}$

formulations	τo	k	n	R^2
AG	39.3 (± 5.3)	19.6 (± 3.9)	0.35 (± 0.03)	0.9838
DCI warm	0.2 (± 0.09)	2.9 (± 0.3)	0.54 (± 0.02)	0.9971
DCI cold	3.7 (± 0.7)	3.0 (± 0.3)	0.51 (± 0.02)	0.9946
DCD	13.2 (± 3.3)	16.8 (± 2.5)	0.29 (± 0.02)	0.9856
PM	7.9 (± 0.4)	1.3 (± 0.1)	0.73 (± 0.01)	0.9982
VC	9.5 (± 3.1)	18.4 (± 2.1)	0.37 (± 0.02)	0.9920
